# Genome-wide analysis of the *EIN3/EIL* family in rye and functional identification of *ScEIL5* in stripe rust resistance

**DOI:** 10.3389/fpls.2026.1791751

**Published:** 2026-03-19

**Authors:** Yarong Wang, Fei Tao, Wenhua Du

**Affiliations:** 1 College of Grassland Science, Key Laboratory of Grassland Ecosystem (Ministry of Education), Sino-U.S. Centers for Grazing Land Ecosystem Sustainability, Gansu Agricultural University, Lanzhou, China; 2 College of Grassland Science, Pratacultural Engineering Laboratory of Gansu Province, Sino-U.S. Centers for Grazing Land Ecosystem Sustainability, Gansu Agricultural University, Lanzhou, China; 3 College of Plant Protection, Gansu Agricultural University, Biocontrol Engineering Laboratory of Crop Diseases and Pests of Gansu Province, Lanzhou, China

**Keywords:** EIN3/EIL, *Secale cereale*, stress response, stripe rust, virus-induced gene silencing (VIGS)

## Abstract

**Introduction:**

Introduction: Rye (*Secale cereale* L.) is a cereal crop with superior overall stress tolerance, particularly prominent resistance to stripe rust, cold, and drought. It thus serves as an important donor of resistance genes for other cereal species. Ethylene-insensitive 3/ethylene-insensitive 3-like (EIN3/EIL) proteins are core transcription factors that transduce ethylene signals into downstream transcriptional cascades. However, their systematic characterization and defense-related functions in rye remain unexplored.

**Methods:**

Here, we performed genome-wide identification of *ScEIL* genes from the rye reference genome, designated them based on chromosomal distribution, and analyzed their gene structure, collinearity, and cis-elements. We also examined the expression profiles of *ScEIL* genes under biotic and abiotic stresses, determined the subcellular localization of *ScEIL5*, and verified its function via VIGS-mediated silencing.

**Results:**

Seven ScEIL genes (*ScEIL1*–*ScEIL7*) were identified. Gene structure analysis revealed a highly conserved N-terminal DNA-binding domain and divergent C-terminal regions. Collinearity analysis showed significant evolutionary divergence of the EIN3/EIL family between monocotyledonous and dicotyledonous plants. *Cis*-element prediction and expression profiling indicated that *ScEIL* genes are involved in rye responses to various stress conditions and exhibit significant tissue specificity, with the lowest expression detected in seeds. *ScEIL5* is localized to the nucleus and showed approximately 4.8-fold higher expression at 48 h post-inoculation with *Puccinia striiformis* f. sp. *tritici* compared with 0 h. VIGS-mediated silencing of *ScEIL5* enhanced stripe rust resistance and reduced fungal biomass.

**Discussion:**

These results suggest that *ScEIL5* acts as a negative regulator of stripe rust resistance in rye. This study not only provides valuable insights for further research on the *EIN3/EIL* gene family, but also offers important clues regarding the role of *ScEIL* genes in rye’s response to stripe rust stress.

## 1 Introduction

Rye (*Secale cereale* L.) is an outcrossing member of the Poaceae family characterized by high dietary fibre, elevated protein content, abundant minerals, and bioactive phenolic compounds. Predominantly cultivated in northern and eastern Europe, it is utilized for grain, forage, and green-manure purposes (Rodehutscord et al., 2016; Torrinha et al., 2019; Ikram et al., 2023). Rye exhibits exceptional tolerance to low temperature, drought, and stripe rust, a characteristic that has drawn extensive attention from researchers to the molecular mechanisms underlying its biotic/abiotic stress tolerance. The species also possesses a large and highly repetitive genome that has only recently been fully sequenced and annotated (Rabanus-Wallace et al., 2021; Bauer et al., 2017).

Ethylene (C_2_H_4_) is a gaseous phytohormone that regulates virtually every phase of the plant life cycle, from seed germination and root hair development to fruit ripening and organ senescence (Ju and Chang, 2015; Guo and Ecker, 2004). The canonical ethylene signalling pathway begins with ethylene perception by a family of endoplasmic reticulum-localized receptors (ETR1, ERS1, EIN4, ETR2, and ERS2) that, in the absence of the hormone, activate the Raf-like kinase CTR1 (Kieber et al., 1993). CTR1 phosphorylates and thereby destabilises the positive regulator EIN2, preventing the nuclear accumulation of the EIN3/EIL transcription factors (Alonso et al., 1999; Ju et al., 2012). Upon ethylene binding, receptor activity is repressed, CTR1 is inactivated and EIN2 is dephosphorylated and proteolytically cleaved to release a C-terminal fragment that migrates to the nucleus and stabilises EIN3/EIL proteins (Wen et al., 2012; Qiao et al., 2012). Stabilised EIN3/EIL then bind directly to the promoter of primary ethylene-response genes such as ERF1, initiating transcriptional cascades that reprogram cellular metabolism and development (Solano et al., 1998).

EIN3 was first identified in *Arabidopsis thaliana* via a screen for ethylene-insensitive mutants that failed to exhibit the triple-response phenotype in etiolated seedlings (Chao et al., 1997). The locus encodes a 628-amino-acid nuclear protein with a highly conserved N-terminal DNA-binding domain (DBD) composed of five α-helices and seven β-sheets that specifically recognises the primary ethylene-response element (PERE; 5’-TGTCTC-3’) and the adjacent GC-rich motif (Solano et al., 1998; Yamasaki et al., 2005). In addition to the DBD, EIN3 contains a proline-rich transcriptional activation domain and a C-terminal region that mediates dimerisation and interaction with the F-box proteins EBF1 and EBF2, which target EIN3 for 26S proteasome-mediated degradation in the absence of ethylene (Wen et al., 2012; Hao et al., 1998; Ohme-Takagi and Shinshi, 1995; Guo and Ecker, 2003; Potuschak et al., 2003). Outside Arabidopsis, EIN3/EIL homologues have been identified in both monocots and dicots, including rice (Mao et al., 2006), maize (Gallie and Young, 2004), wheat (Duan et al., 2013), tomato (Tieman et al., 2001), petunia (Shibuya et al., 2004), and carnation (Iordachescu and Verlinden, 2005).

The functional versatility of EIN3/EIL proteins stems from their ability to integrate ethylene signalling with other hormone pathways, including gibberellin (An et al., 2012), jasmonic acid (Zhu et al., 2011) and salicylic acid (Chen et al., 2009). In Arabidopsis, EIN3 directly represses SID2 expression, thereby attenuating SA biosynthesis and modulating the trade-off between growth and defence (Chen et al., 2009). Conversely, EIN3 and EIL1 synergistically activate HLS1 transcription in cooperation with GA signalling to promote apical hook formation in etiolated seedlings (An et al., 2012). During leaf senescence, EIN3 induces ORE1 expression by suppressing miR164, accelerating chlorophyll degradation and nutrient remobilisation (Li et al., 2013). Similar pleiotropic roles have been reported in crops: overexpression of *OsEIL1* in rice enhances ethylene sensitivity and promotes internode elongation in deep-water conditions (Hattori et al., 2009), whereas silencing of *LeEILs* in tomato extends flower longevity by delaying petal senescence (Iordachescu and Verlinden, 2005). Beyond developmental control, EIN3/EIL proteins play pivotal roles in biotic/abiotic stress adaptation (Chen et al., 2005; Kazan, 2015; Tao et al., 2015). In wheat, *TaEIL1* acts as a negative regulator of stripe rust resistance by suppressing pathogenesis-related (PR) gene expression (Duan et al., 2013), whereas in cotton, *GhEIL3* enhances drought tolerance by activating ROS-scavenging enzymes and ABA-responsive genes (Zhang et al., 2023). Under salt stress, Arabidopsis EIN3 directly binds to the promoter of ESE1 (ETHYLENE AND SALT-INDUCIBLE ERF1) to activate the expression of peroxidases and superoxide dismutases, thereby mitigating oxidative damage (Zhang et al., 2011). In rice, *OsEIL1* positively regulates the expression of *OsAPX2* and *OsSOD1* under drought and high-salinity conditions (Cao et al., 2006). *MdEILs* in apples regulate fruit ripening mechanisms and respond to cold treatment (Feng et al., 2025). Recently, Liu et al. (2019) showed that mulberry *MnEIL3* confers both salt and drought tolerance by modulating ethylene biosynthetic genes and ABA signalling. Collectively, these studies have demonstrated the functional diversity of EIN3/EIL proteins.

Given rye exhibits distinctive stress tolerance, further elucidation of its *EIN3/EIL* gene family may facilitate identification of novel molecular mechanisms underlying disease resistance. In this study, we employed bioinformatics to identify and characterize rye *EIN3/EIL* gene family members. Their responses to biotic and abiotic stresses were quantified by qRT-PCR, and tissue-specific expression patterns were analyzed using publicly available transcriptome datasets. *ScEIL5* was significantly upregulated upon stripe rust infection, and VIGS-mediated silencing confirmed its function as a negative regulator of disease resistance. These findings provide valuable insights into the *EIN3/EIL* gene family and its biological function in rye.

## 2 Materials and methods

### 2.1 Genome-wide identification of the *EIN3/EIL* gene family and biochemical characterization of ScEIL proteins in rye

To identify *EIN3/EIL* family members in rye, Arabidopsis EIN3/EIL protein sequences were retrieved from TAIR (https://www.arabidopsis.org) and used as BLAST queries against the rye reference genome (Ensembl Plants; https://www.ncbi.nlm.nih.gov/datasets/genome/GCA_902687465.1/). Candidate loci were refined by HMMER3.0 searches (Pfam PF04873) and validated for the presence of the EIN3/EIL conserved domain using the Conserved Domain Database (CDD; https://www.ncbi.nlm.nih.gov/Structure/cdd/) (Marchler-Bauer et al., 2017).

Physicochemical properties of ScEIL proteins were predicted with ExPASy ProtParam (https://web.expasy.org/protparam/) (Artimo et al., 2012), and subcellular localization was inferred with Cell-PLoc 2.0 (http://www.csbio.sjtu.edu.cn/bioinf/Cell-PLoc-2/).

### 2.2 Chromosomal localization and gene duplication events

The chromosomal start and end location information of *ScEIL* genes was extracted from gene annotation files, and their physical locations were mapped using TBtools software. Intraspecific synteny of the rye *EIN3/EIL* family was analyzed via the One-Step MCScanX function in TBtools, and the results were visualised in the software. Furthermore, One Step MCScanX-Super Fast was utilised to visualise interspecific collinearity analysis of the *EIN3/EIL* family in rye and other species (Chen et al., 2023).

### 2.3 Construction of the phylogenetic tree and analysis of the gene structure

Phylogenetic trees were constructed with MEGA 7.0 using the Neighbor-Joining method (1,000 bootstrap replicates) (Kumar et al., 2016). Conserved motifs were identified with MEME (https://meme-suite.org; max = 12) and visualised together with exon-intron structures via TBtools. Multiple sequence alignment was performed with ClustalX (Thompson et al., 1997) and edited in GeneDoc (Larkin et al., 2007). Tertiary structures were modeled with SWISS-MODEL (https://swissmodel.expasy.org/) using the best Protein Data Bank (PDB) template (Waterhouse et al., 2018).

### 2.4 Cis- acting elements and functional prediction

Using PlantCARE (https://bioinformatics.psb.ugent.be/webtools/plantcare/html/) database of *EIN3/EIL* genes promoter sequences (translation initiation site upstream 2 kbp) of cis regulatory elements (Lescot et al., 2002). The regulatory functions of the predicted *cis-acting* elements were subjected to a classification and statistical analysis.

### 2.5 Subcellular localization

The CDS sequence of the *ScEIL5* gene was amplified by specific primers, and the product was ligated to the pCAMBIA1302-GFP vector to obtain 35S:ScEIL5-GFP. HY5 (nuclear localization protein) was cloned and ligated into pCAMBIA1302-mCherry to obtain 35S:HY5-RFP (Wang et al., 2021). The fusion carrier was introduced into 4-week-old tobacco leaves for instantaneous expression. After infiltration for 48 h, it was observed using a confocal laser scanning microscope (Olympus, Japan). The detailed acquisition parameters were set as follows: excitation wavelength for GFP was 488 nm, emission wavelength was 500–550 nm; excitation wavelength for RFP was 552 nm, emission wavelength was 570–620 nm.

### 2.6 Plant materials and treatments

Seeds of ‘Gannong NO. 2’ rye provided by the Grassland Science College of Gansu Agricultural University was used as experimental material.

Seeds were surface-sterilized in 1% sodium hypochlorite solution for 5 minutes and rinsed three times with sterile distilled water. Sterilized seeds were germinated on moist filter paper at 25 °C for 3 days. For abiotic stress treatments, germinated seedlings were transferred to Hoagland nutrient solution (pH 5.8) and hydroponically cultured under controlled conditions (16 h light/8 h dark cycle; light intensity: 250 μmol/m^2^/s; temperature: 25 ± 2 °C; relative humidity: 60% ± 5%) until the three-leaf stage. Uniformly growing seedlings were selected for subsequent stress treatments. Drought stress: Seedlings were transferred to a nutrient solution supplemented with 15% polyethylene glycol (PEG-6000) to simulate drought stress conditions. Low temperature stress: Seedlings were transferred to an incubator maintained at 4 °C, with all other environmental conditions kept unchanged. Leaves were collected at 0 h, 6 h, 12 h, and 24 h post-treatment for each stress. For biotic stress treatment, germinated seeds were sown in 15 cm-diameter flower pots (10 seedlings per pot) and grown under the aforementioned controlled conditions until the three-leaf stage. The biotic stress, *Puccinia striiformis* f. sp. *tritici* (*Pst*), was applied in a uniformly to dewaxed leaves (dewaxed by gentle manual friction with fingers) at 9-12 °C. Inoculated plants were maintained in a high-humidity environment to facilitate pathogen infection. Leaves were harvested at 0, 24, 48 and 72 hours post-inoculation (hpi). Each treatment included three biological replicates. All samples were immediately frozen in liquid nitrogen and stored at −80 °C.

### 2.7 Gene expression analysis

Tissue-specific expression of *ScEIL* genes in seeds, roots, stems, and leaves was analyzed based on RNA-seq data retrieved from the National Center for Biotechnology Information (NCBI) Sequence Read Archive (SRA) database (accession no. PRJNA680499). Fragments per kilobase of transcript per million mapped reads (FPKM) was used to quantitatively calculate gene expression levels (Trapnell et al., 2009).

Total RNA was extracted using the Plant RNA Extraction Kit (TIANGEN, China) according to the manufacturer instructions. The reverse transcription of the RNA samples was conducted using the SweScript All-in-One RT SuperMix for qPCR (One-Step gDNA Remover) kit, following the manufacturer protocol. Specific primers were designed based on the gene sequence using the Primer 5.0 software. The primers are listed in **Supplementary Table 1**. The expression of *ScEIL* genes was quantified by quantitative reverse transcription polymerase chain reaction (qRT-PCR) on a LightCycler^®^ 96 system. Three technical replicates were run per biological sample. The reaction system included 10 μL of SYBR Premix Ex Taq (2×), 0.4 μL of each primer (10 μM), 2 μL of template (approximately 100 ng/μL), and 7.2 μL of ddH_2_O, resulting in a total volume of 20 μL. The qRT-PCR program was as follows: pre-denaturation at 95 °C for 30 s, denaturation at 95 °C for 15 s, annealing/extension of the primer at 60 °C for 30 s, with fluorescence signal collection. A total of 35 cycles were performed. Actin (GenBank Accession: FJ032189.1) was used as the internal parameter to calculate the relative expressions via the Ct (2^−ΔΔCt^) method (Livak and Schmittgen, 2001; Ren et al., 2023).

### 2.8 Virus-induced gene silencing

The function of *ScEIL5* in rye was analyzed using a virus-induced gene silencing (VIGS) approach. Plasmid construction for gene silencing was performed as previously described by Holzberg et al. (2002). The specific primers were designed to amplify gene-specific fragments of *ScEIL5* (**Supplementary Table 1**), incorporating PacI and NotI restriction sites. The amplified fragments replaced the phytoene desaturase (PDS) fragment in the γ-PDS positive control vector, resulting in the construction of two vectors, Barley stripe mosaic virus (BSMV):γ-*ScEIL5*-1as and BSMV:γ-*ScEIL5*-2as. BSMV:α and BSMV:γ vectors were digested with MluI, while BSMV:β was digested with SpeI. Subsequently, both BSMV:γ-PDS, BSMV:γ-*ScEIL5*-1as and BSMV:γ-*ScEIL5*-2as were digested with BssHII. The digested fragments were recovered and purified. Using these linearized products as templates, *in vitro* transcription was performed with the T7 RiboMAX kit (Promega, USA). A mixture of 0.5 uL BSMV:α, BSMV:β, and BSMV:γ (or BSMV:γ-PDS, BSMV:γ-*ScEIL5*-1as, and BSMV:γ-*ScEIL5*-2as) was combined with 8.5 uL of FES buffer and friction inoculation onto the second leaf at the three-leaf stage. The inoculation method followed (Panwar and Kanyuka, 2022). FES buffer was used as a negative control, and BSMV:γ-PDS served as a positive control. Plants were incubated at 25 °C for 12 days until photobleaching symptoms appeared on BSMV: PDS-inoculated leaves. Once photobleaching was observed, the fourth leaf was inoculated with *Pst* CYR34. The inoculated leaves were collected at 0, 24, 72, and 120 hpi for silencing efficiency analysis. Additional samples were harvested at 24, 72 and 120 hpi for microscopic examination.

### 2.9 Histological analysis

Using the method described by Redkar et al. (2018), *Pst*-inoculated leaves were first subjected to decolorization treatment in ethanol:acetic acid (1:1, v/v). Subsequently, cleared tissues were washed with 50 mM Tris-HCl (pH 7.4) and incubated with wheat germ agglutinin Alexa-488 (product number W11261, Thermo Fisher Scientific). Finally, samples were photographed under an Olympus BX53 microscope. Infection areas, hyphal lengths, and haustorial mother cell numbers were quantified using CellSens Entry software.

For H_2_O_2_ detection, leaves were immersed in a 3,3’-diaminobenzidine (DAB) solution (1 mg/mL, pH 3.8) and incubated under light for 6 h. After destaining in ethanol:acetic acid (1:1, v/v), samples were photographed under an Olympus BX53 microscope. DAB-stained areas were quantified with CellSens Entry.

### 2.10 Statistical analysis

All data are presented as the mean ± standard deviation (SD) from at least three independent biological replicates. Statistical analyses were performed using IBM SPSS 20.0 Statistics software. Normality and homogeneity of variances were assessed using Shapiro–Wilk and Levene’s tests, respectively. Since the assumptions were not met, the nonparametric Kruskal–Wallis H test was performed. *Post hoc* multiple comparisons were conducted using Dunn’s test with Bonferroni correction to identify pairwise differences. Statistical significance was set at *p* < 0.05, and significant differences are indicated by different lowercase letters.

## 3 Results

### 3.1 Genome-wide identification, chromosome localization and gene structure analysis of *ScEILs* in rye

**Figure 1:**
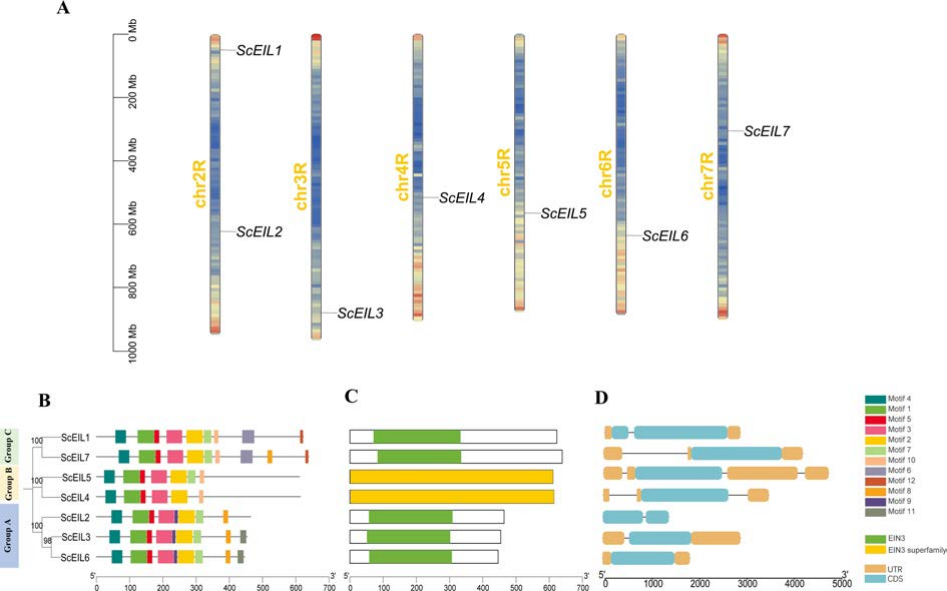
Analysis of chromosome localization and gene structure of the *ScEIL* genes. **(A)** Distribution of *ScEILs* on rye chromosomes. The scale bar indicates chromosome lengths, with regions of higher gene density shown in red and regions of lower gene density shown in blue. **(B)** Conserved motifs in ScEIL proteins identified by MEME. Twelve motifs are displayed in different colors. **(C)** Conserved protein domains. Green boxes indicate EIN3 domains; yellow boxes indicate EIN3 superfamily domains. **(D)** Exon-intron organization of *ScEIL* genes.

To identify *EIN3/EIL* genes in rye, we used *A. thaliana* (*AtEIL*, n=6) and wheat (*TaEIL*, n=21) protein sequences as queries to search the rye genome. The obtained sequences were then extracted using the HMM. Subsequently, candidate gene sequences were filtered through the Conserved Domain Database (CDD) to eliminate redundant sequences. Ultimately, seven *ScEIL* genes were identified. According to the gene annotation information file (GFF3), these seven *ScEIL* genes were located on six chromosomes (**Figure 1A**). Based on their chromosomal order, these genes were named *ScEIL1* to *ScEIL7*. *ScEIL1* and *ScEIL2* were located on the same chromosome (chr2R), while the remaining five *ScEIL* genes were distributed on chr3R, chr4R, chr5R, chr6R, and chr7R, respectively.

Twelve conserved motifs were identified based on MEME online analysis. Motifs 1–5 were located at the N-terminus (**Figure 1B**) and are present in all seven ScEIL proteins. This indicated that the EIN3/EIL protein sequences of rye were highly conserved at the N-terminus, a feature that is also present in EIN3/EIL proteins of other species. Notably, ScEIL proteins that are closely related on the phylogenetic tree have similar structural compositions, suggesting that they may have functional redundancy. Some of these motifs were found to be unique to members of group A (motif 11) or group C (motifs 6 and 12), suggesting that these motifs may have unique functions. In addition, protein domain analysis showed that groups A and C contained the EIN3-specific domain, which is a unique domain defining the EIN3 subfamily, whereas group B contained the EIN3 superfamily domain, which represents a conserved domain shared by the entire *EIN3/EIL* family (**Figure 1C**). To further explore the gene structure of *ScEILs*, the exon-intron organization of these genes was analyzed. As shown in **Figure 1D**, the number and length of introns varied substantially among *ScEIL* genes. Group B contained the highest number of introns. Specifically, *ScEIL4* harbored two introns and *ScEIL5* contained three introns.

### 3.2 Phylogenetic analysis and physicochemical properties of the ScEIL proteins

**Figure 2:**
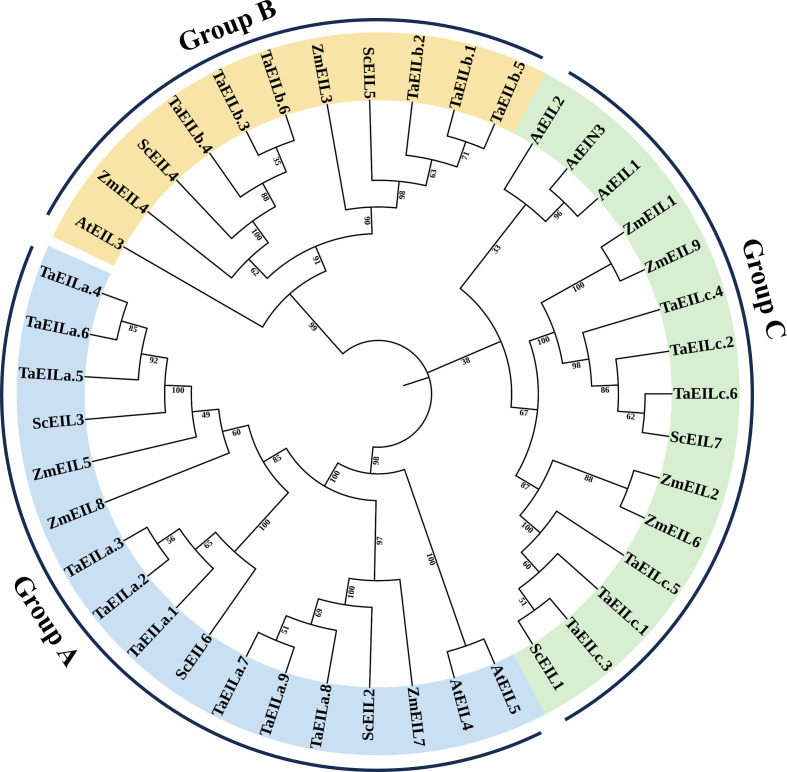
Phylogenetic tree of EIN3/EIL proteins from rye (ScEILs), wheat (TaEILs), maize (ZmEILs), and *Arabidopsis* (AtEILs) constructed using the neighbor-joining method with 1000 bootstrap replicates in MEGA 7.0.

To investigate the evolutionary relationships of EIN3/EIL proteins, a phylogenetic tree was constructed using 43 amino acid sequences from *Triticum aestivum* (TaEILs, n=21), *Zea mays* (ZmEILs, n=9), *A. thaliana* (AtEILs, n=6), and *S. cereale* (ScEILs, n=7). The phylogenetic tree clearly clustered the EIN3/EIL proteins into three distinct clades (group A, B, and C), with each clade comprising members from both monocot and dicot species (**Figure 2**). The close clustering of ScEILs with TaEILs illustrated the tight kinship between rye and wheat and indicated strong functional conservation, consistent with previous classifications in Arabidopsis and wheat.

Analysis of the physicochemical properties revealed that ScEIL proteins ranged in length from 446 amino acids (ScEIL2 and ScEIL6) to 639 amino acids (ScEIL7) (**Table 1**). Molecular weights ranged from 49.29 kDa (ScEIL2) to 70.89 kDa (ScEIL7). Isoelectric points ranged from 5.12 (ScEIL7) to 5.89 (ScEIL5), indicating that these proteins are acidic. Hydropathy indices ranged from -0.547 (ScEIL1) to -0.721 (ScEIL7), indicating that these proteins are hydrophilic. In addition, ScEIL4 was identified as a stable protein (instability index < 40), while the other proteins were unstable (instability index > 40). Protein stability here refers to predicted structural stability and resistance to degradation. These values represent in silico predictions of protein structural stability rather than experimentally determined functional stability. Subcellular localization prediction analysis indicated that all members of the ScEIL protein family were localized in the nucleus.

**Table 1:** The physicochemical properties of the ScEIL proteins.

Name	ID	Length (aa)	WM (kDa)	pI	Gravity	Instability index	Subcellular location
ScEIL1	SECCE2Rv1G0071970.1	623	67.68	5.68	-0.547	62.46	Nucleus.
ScEIL2	SECCE2Rv1G0105520.1	446	49.29	5.52	-0.654	52.63	Nucleus.
ScEIL3	SECCE3Rv1G0202840.1	454	50.62	5.3	-0.715	62.09	Nucleus.
ScEIL4	SECCE4Rv1G0244500.1	614	68.09	5.52	-0.649	37.44	Nucleus.
ScEIL5	SECCE5Rv1G0334180.1	611	68.25	5.89	-0.685	45.76	Nucleus.
ScEIL6	SECCE6Rv1G0414700.1	446	49.3	5.52	-0.654	57.69	Nucleus.
ScEIL7	SECCE7Rv1G0482440.1	639	70.89	5.12	-0.721	51.63	Nucleus.

WM, Molecular weight; pI, Isoelectric point; Gravity: Grand average of hydropathy.

### 3.3 Gene duplication and collinearity analysis of *ScEILs*

**Figure 3:**
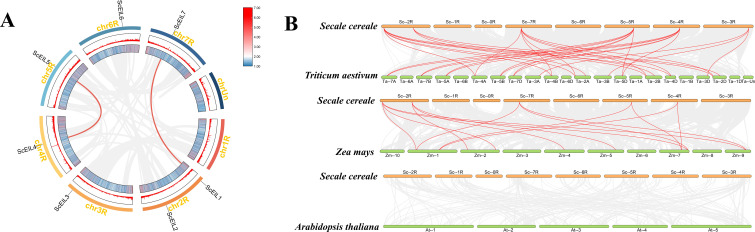
Duplication and collinearity analysis of *ScEIL* genes. **(A)** Segmental duplication of *ScEIL* genes in rye. Paralogous gene pairs are connected by red lines; heat maps on chromosomes indicate gene density. **(B)** Collinearity analysis of *ScEIL* genes with *T. aestivum*, *Z. mays*, and *A. thaliana*. Gray lines show syntenic blocks; red lines highlight *ScEIL* orthologous pairs.

To investigate the expansion mechanism of the *ScEIL* gene family in rye, we analyzed gene duplication events. We identified two segmental duplication events: one between *ScEIL4* and *ScEIL5* (on chr4R and chr5R), and another between *ScEIL1* and *ScEIL7* (on chr2R and chr7R) (**Figure 3A**). These duplicated sequence pairs, belonging to groups B and C, further indicate that fragment duplication may be a major driving force for the expansion of the *ScEIL* gene family in rye, providing a genetic basis for functional divergence and evolution of this gene family.

To investigate the evolutionary conservation and divergence of *ScEIL* genes in rye, we conducted a collinearity analysis of *EIN3/EIL* genes in rye, wheat, maize, and *Arabidopsis* (**Figure 3B**). The results revealed multiple homologous gene pairs between rye and both wheat and maize, indicating that *EIN3/EIL* genes in these species exhibit a high degree of evolutionary conservation. This reflects their close phylogenetic relationship within the grass family. During the evolution of the *EIN3/EIL* gene family, some ancestral gene characteristics may have been retained. No homologous gene pairs were identified between rye and *Arabidopsis EIN3/EIL* genes, reflecting that due to the long evolutionary distance between these species, the conservation of *EIN3/EIL* genes has been significantly reduced.

### 3.4 Sequence and structural characterization of the DNA-binding domain in *ScEIL* transcription factors

**Figure 4:**
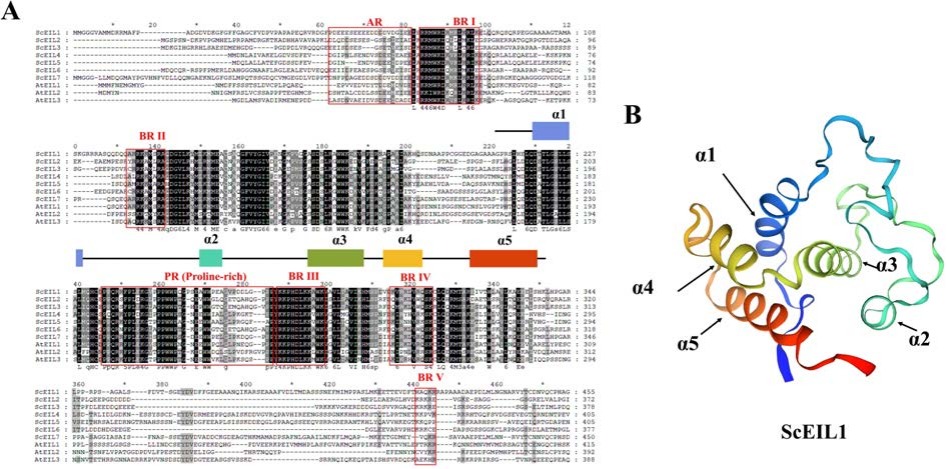
Sequence alignment and three-dimensional structure of ScEIL proteins. **(A)** Multiple sequence alignment of EIN3/EIL proteins from rye and *Arabidopsis*. AR, Acidic region; BR, Basal regions. Red boxes indicate conserved regions. Black, dark gray, and light gray shading indicates 100%, 80%, and 60% similarity, respectively. The black line marks the DNA-binding domain (DBD); colored regions mark α-helices. **(B)** Tertiary structure of ScEIL1. Arrows indicate five α-helices; with the N- and C-terminal regions colored blue and red, respectively.

Multiple sequence alignment of seven ScEIL and three AtEIL proteins revealed seven conserved regions (**Figure 4A**). Five basal regions (BRI-BRV), one acidic region (AR), and one proline-rich domain (PR) were identified. The DBD region exhibited high conservation, particularly in the α1, α3, and α4 helical regions. Key amino acid residues (such as Arg and Lys) were completely conserved, which may be crucial for DNA-binding activity. Additionally, the C-terminal PR region of ScEIL proteins showed significant variation, which may be related to specific regulation of their transcriptional activation functions.

Homology modeling showed that the DNA-binding domain (DBD) of ScEIL proteins consists of five α-helices (**Figure 4B**). These helical structures form a characteristic three-dimensional fold that is expected to mediate specific binding to target DNA sequences.

### 3.5 Cis-acting element analysis

**Figure 5:**
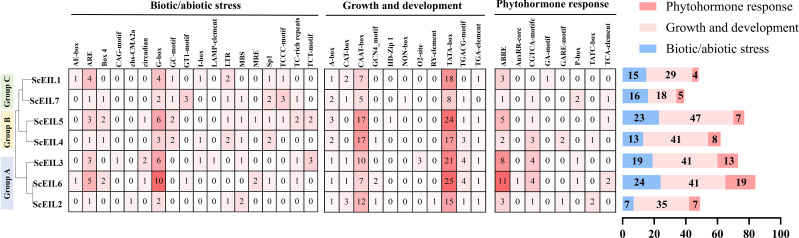
*Cis-actin*g elements in *ScEIL* gene promoters. Elements are categorized into three groups: biotic/abiotic stress, growth and development, and phytohormone responses. Numbers in boxes indicate element counts. The bar chart summarizes element numbers per gene.

Analysis of *cis-acting* elements in *ScEIL* gene promoters revealed the presence of elements involved in responses to biotic/abiotic stress, growth and development, and phytohormones (**Figure 5**). Among these, elements associated with plant growth and development were the most abundant (n = 252), followed by those related to biotic/abiotic stress (n = 117), while phytohormone-responsive elements were the least abundant (n = 63).

Among the 18 biotic/abiotic stress-related elements, G-box and ARE-involved in light responsiveness and antioxidant defense—were identified. The LTR element, associated with low-temperature stress responses, was also present. MBS, related to drought induction, and TC-rich repeats, involved in defense and pathogen stress responses, were additionally detected.

Among the 11 growth and development-related elements, the core promoter element TATA-box, involved in transcription initiation complex assembly, was included. CAAT-box, which enhances transcriptional efficiency; GCN4_motif, involved in endosperm development and amino acid metabolism; and HD-Zip 1, related to light responsiveness and organ development, were also identified.

Among the eight phytohormone-responsive elements, ABRE, responsive to abscisic acid (ABA), was present. AuxRR-core, responsive to auxin; TGA-element, involved in salicylic acid signaling; GA_motif, responsive to gibberellin; and P-box, regulating seed germination and stem elongation, were also identified.

### 3.6 Gene expression levels under biotic/abiotic stress

**Figure 6:**
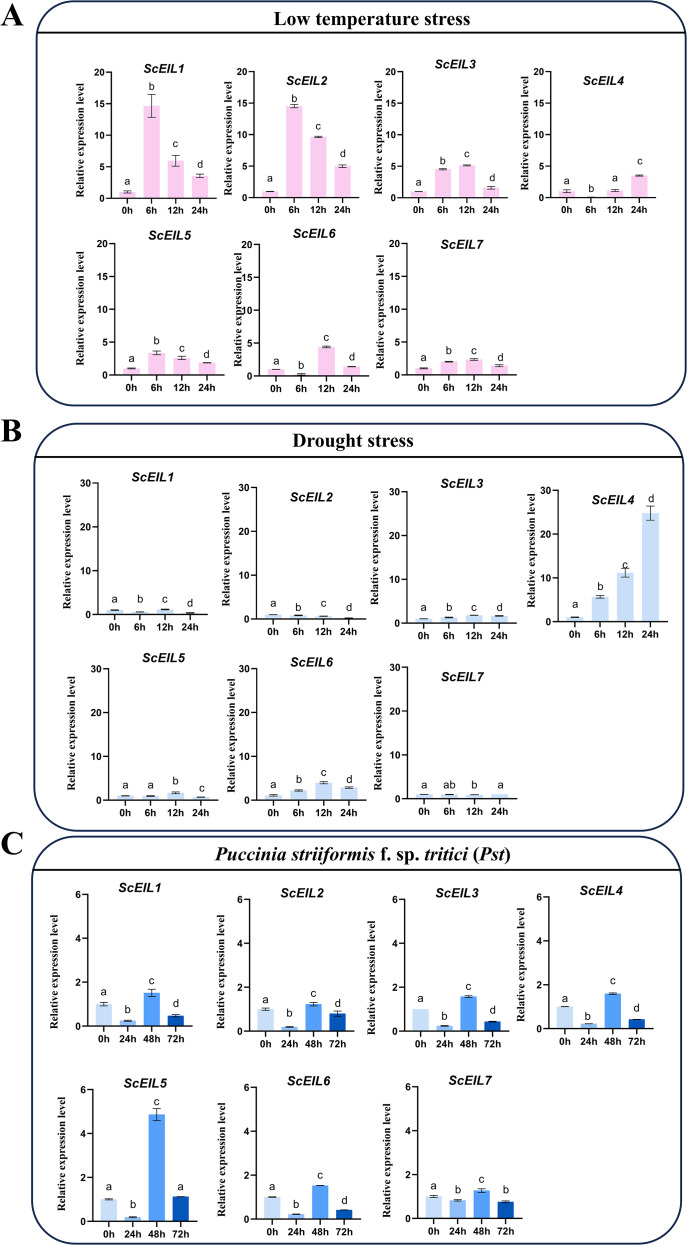
Expression analysis of *ScEIL* genes under stress conditions. **(A)** Low-temperature stress. **(B)** Drought stress. **(C)** *Puccinia striiformis* f. sp. *tritici* (*Pst*) infection. Biological replicates (n = 3): each replicate represents an independent rye plant; 3 technical qRT-PCR replicates were performed per plant sample, and cycle threshold (Ct) values were averaged per plant for analysis. Data are means ± SD. Different letters indicate significant differences at *p* < 0.05 (Kruskal–Wallis and Dunn’s test).

To elucidate the potential roles of *ScEIL* genes in rye under abiotic stress, we analyzed their expression patterns under drought and low-temperature stress using qRT-PCR. Under low-temperature stress, *ScEIL* genes exhibited diverse expression profiles in leaves. For instance, *ScEIL1*, *ScEIL2*, *ScEIL5*, and *ScEIL7* were significantly upregulated at 6 h, with expression levels of *ScEIL1* and *ScEIL2* increasing 14.7- and 14.5-fold compared to 0 h, respectively (**Figure 6A**). This strong induction suggests that these genes may play key roles in regulating rye response to low temperature stress. In contrast, *ScEIL3* and *ScEIL7* showed continuous upregulation but were significantly downregulated at 24 h.

Under drought stress, *ScEIL* genes displayed distinct expression dynamics. *ScEIL3*, *ScEIL5*, and *ScEIL6* were initially upregulated but declined at 24 h (**Figure 6B**). *ScEIL2* showed a gradual downregulation throughout the treatment period. In contrast, *ScEIL4* expression steadily increased over 24 h, reaching 24.8-fold induction at 24 h. These differential expression patterns suggest that *ScEIL* genes may contribute specifically and distinctly to drought stress responses.

To investigate the role of *ScEIL* genes under biotic stress, we analyzed their expression following inoculation with *Puccinia striiformis* f. sp. *tritici* (*Pst*) CYR34. The results revealed a general trend of initial downregulation, followed by upregulation and then a second downregulation (**Figure 6C**). Notably, *ScEIL5* expression was significantly induced at 48 h, increasing 4.8-fold compared to 0 h. These findings suggest that *ScEIL5* plays a crucial role in rye defense response to stripe rust.

### 3.7 The tissue-specific expression of the *EIN3/EIL* gene family in rye and subcellular localization analysis of ScEIL5

**Figure 7:**
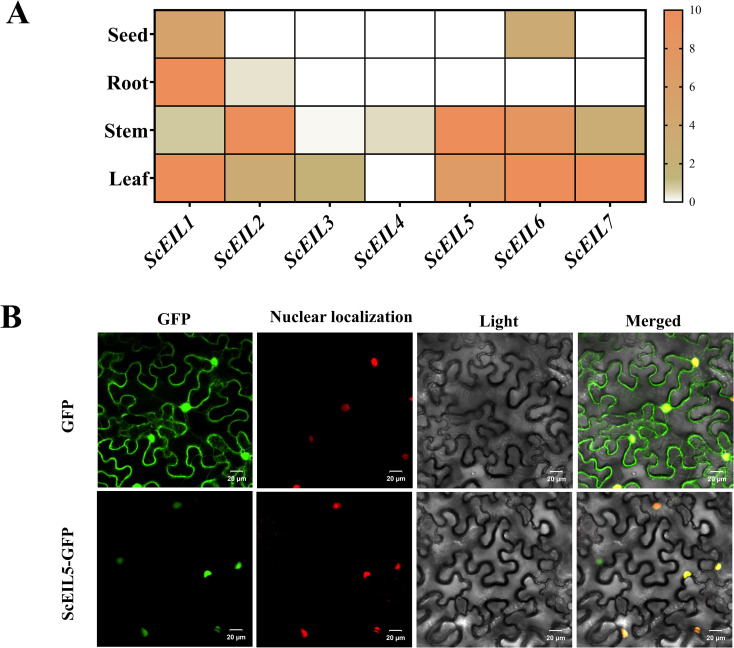
Tissue-specific expression and subcellular localization of *ScEIL5*. **(A)** Tissue-specific expression pattern of *ScEIL*. White: non-detectable. Higher values on the color scale represent greater gene expression abundance. **(B)** Subcellular localization of *ScEIL5* in tobacco leaf cells. The CDS of *ScEIL5* was cloned into pCAMBIA1302-GFP to generate 35S:ScEIL5-GFP. The nuclear marker HY5 was constructed into pCAMBIA1302-mCherry to produce 35S:HY5-RFP. The two constructs were co-transformed into 4-week-old tobacco leaves for transient expression. Fluorescence signals were observed under a confocal laser scanning microscope (Olympus, Japan) at 48 h after infiltration. Scale bar: 20 μm. Channels: GFP (green, ScEIL5), RFP (red, nuclear marker HY5), Merged, and Bright field.

We analyzed the tissue-specific expression patterns of *ScEIL* genes by interrogating a public rye transcriptome database, examining data from seeds, roots, stems, and leaves (**Figure 7A**). The results showed that *ScEIL* genes exhibited significant tissue-specific expression. *ScEIL1* was predominantly expressed in roots and leaves. *ScEIL7* was predominantly expressed in leaves. *ScEIL2* showed high expression in stems. *ScEIL5* and *ScEIL6* were also highly expressed in stems and leaves. Expression of *ScEIL* genes in seeds was generally low, with only *ScEIL1* and *ScEIL5* showing detectable levels. This tissue-specific expression pattern suggests that *ScEIL* genes may play differentiated regulatory roles in the growth, development, or physiological processes of different rye tissues.

Previous experiments have shown that *ScEIL5* may play an important role in resistance to *Pst* infection. Therefore, we investigated the subcellular localization of *ScEIL5*. A ScEIL5-GFP fusion expression vector was constructed, with an empty GFP vector used as a control. Subcellular localization was observed following transient transformation of tobacco leaves. The results showed that the green fluorescence signal of the ScEIL5-GFP fusion protein was mainly concentrated in the nucleus and completely overlapped with the red fluorescence signal of the nuclear marker (**Figure 7B**). This indicated that ScEIL5 was a nuclear-localized protein, consistent with its function as a transcription factor that exerts gene regulatory activity in the nucleus.

### 3.8 Inhibiting *ScEIL5* can enhance the resistance of rye to *Pst*

**Figure 8:**
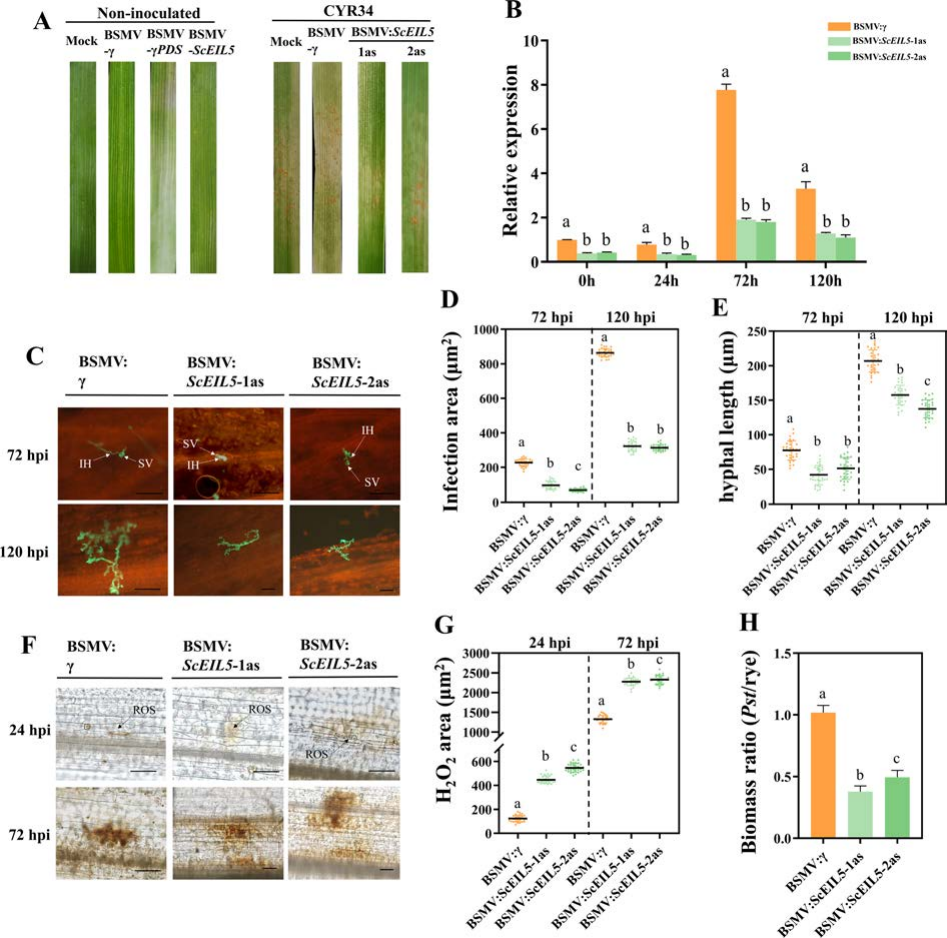
*ScEIL5*-silencing enhances rye resistance to *Puccinia striiformis* f. sp. *tritici* (*Pst*) CYR34. **(A)** Following 12 days of VIGS, typical photobleaching symptoms were evident on the treated rye leaves. Subsequently, the leaves were inoculated with *Pst* CYR34, and phenotypic responses were systematically documented at 16 dpi. **(B)** The transcriptional level of *ScEIL5* was quantified by qRT-PCR to assess the gene silencing efficiency. Biological replicates (n = 3): each replicate represents an independent rye plant; 3 technical qRT-PCR replicates were performed per plant sample, and cycle threshold (Ct) values were averaged per plant for analysis. Data are means ± SD. **(C)** Leaf samples inoculated with *Pst* CYR34 were collected at 72 and 120 hpi. The samples were stained with wheat germ agglutinin conjugated to Alexa 488 followed by fluorescence microscopy observation. SV, substomatal vesicle; IH, infection hypha. Scale bar: 50 μm. **(D)** Average hyphal infection areas were measured in leaf samples collected at 72 and 120 hpi after inoculation with *Pst* CYR34. Biological replicates (n = 3): each replicate represents an independent rye plant; 10 technical subsamples (microscopic fields) were measured per plant. Data are presented as individual data points with means ± SD. **(E)** Hyphal length from the base of substomatal vesicles to hyphal tips. Biological replicates (n = 3): each replicate represents an independent rye plant; 10 technical subsamples (microscopic fields) were measured per plant. Data are presented as individual data points with means ± SD. **(F)** H_2_O_2_ accumulation visualized by DAB staining. **(G)** ROS region area in *Pst* CYR34-inoculated leaves. Biological replicates (n = 3): each replicate represents an independent rye plant; 10 technical subsamples (microscopic fields) were measured per plant. Data are presented as individual data points with means ± SD. **(H)** *Ps*t/rye biomass ratio determined by qRT-PCR, normalized to *ScActin* and *PstEF*. Biological replicates (n = 3): each replicate represents an independent rye plant; 3 technical qRT-PCR replicates were performed per plant sample, and Ct values were averaged per plant for analysis. Different letters indicate significant differences at *p* < 0.05 (Kruskal–Wallis and Dunn’s test).

To investigate the role of *ScEIL5* in rye resistance to stripe rust, we employed virus-induced gene silencing (VIGS) to knock down *ScEIL5* expression. Two independent VIGS constructs (BSMV: *ScEIL5*-1as and BSMV: *ScEIL5*-2as) targeting distinct regions of *ScEIL5* were designed and analyzed separately in this study. Viral infection was confirmed by photobleaching in BSMV: PDS-inoculated plants at 12 days post-inoculation (dpi) (BSMV: Barley stripe mosaic virus; PDS: Phytoene desaturase) (**Figure 8A**). Subsequently, all BSMV-inoculated plants were challenged with *Pst* CYR34. Silencing efficiency was assessed by qRT-PCR, which revealed that both independent VIGS constructs (BSMV:*ScEIL5*-1as and BSMV:*ScEIL5*-2as) significantly reduced *ScEIL5* transcript levels compared with the BSMV:γ empty vector control at 0, 24, 72, and 120 hours post-inoculation (hpi) with *Pst* (**Figure 8B**). The silencing efficiency ranged from 55%–77% for BSMV:*ScEIL5*-1as and 58%–77% for BSMV:*ScEIL5*-2as, respectively (**Figure 8B**). Both constructs exhibited high and consistent silencing efficiency across all time points, further confirming the specificity of gene silencing. At 16 dpi with *Pst*, phenotypic analysis showed a significant reduction in spore production on *ScEIL5*-silenced leaves compared with the control (**Figure 8A**). Furthermore, both infection area and hyphal length were significantly smaller in silenced leaves than in the control at 72 and 120 hpi (**Figures 8C–E**). Histochemical staining with DAB detected significantly greater H_2_O_2_ accumulation in *ScEIL5*-silenced plants than in the control at 24 and 72 hpi (**Figures 8F, G**). Consistent with these findings, fungal biomass was also significantly lower in *ScEIL5*-silenced plants than in the control (**Figure 8H**). Collectively, our results demonstrate that silencing *ScEIL5* enhances resistance to stripe rust in rye, suggesting that *ScEIL5* functions as a negative regulator of plant defense.

## 4 Discussion

The *EIN3/EIL* gene family has been extensively studied in numerous monocotyledonous plants, including maize (Jyoti et al., 2021), wheat (He et al., 2020), and rice (Aluko et al., 2024), as well as in various dicotyledonous species such as *Medicago sativa* (Su et al., 2024) and poplar (Liu et al., 2022). Despite the publication of a complete rye genome sequence (Bauer et al., 2017), the *EIN3/EIL* family in rye remains poorly characterized. In this study, we identified seven members of this transcription factor (TF) family from the rye genome. Phylogenetic analysis grouped these ScEIL proteins into three clades (A, B, and C), with group A containing the most members—a pattern similar to the clustering of *TaEILs* in wheat (He et al., 2020). Gene structure analysis revealed conserved motifs in the N-terminal region but divergent motifs in the C-terminal region, suggesting that the less conserved C-terminus may determine functional specificity. The EIN3/EIL proteins are nuclear-localised DNA-binding proteins and may be involved in a complex network of primary and secondary metabolic pathways in plants (Yamasaki et al., 2005). The conserved domains BRIII, BRIV, and PR contain amino acid residues essential for DNA binding and signal transduction. Collinearity analysis revealed a more conserved evolutionary relationship between rye *EIN3/EIL* genes and monocotyledonous wheat and maize, but no collinearity with dicotyledonous *A. thaliana*. This suggests that the *EIN3/EIL* family exhibits greater evolutionary divergence between monocotyledonous and dicotyledonous plants.

The *cis-acting* elements of the gene promoter region are the basic functional elements of the complex regulatory network of gene response (Xu et al., 2024). *Cis-*acting elements are divided into three categories: biotic/abiotic stress, plant growth and development, and plant hormone response. The composition and number of *cis-acting* elements vary among genes, enabling them to perform distinct biological functions. *Cis-acting* element prediction revealed that the promoter region of *ScEIL* genes were rich in *cis-acting* elements involved in various reactions, including 18 elements involved in biotic/abiotic stress, 11 elements involved in plant growth and development, and 8 elements involved in phytohormone response. This suggests that *ScEILs* may be involved in multiple stress responses. Among the components involved in biotic/abiotic stress, the number of G-box elements was the highest, consistent with the results of wheat. The G-box is a pervasive regulatory DNA element in plants that has been demonstrated to induce drought tolerance by forming a complex with the Basic leucine zipper (bZIP) protein (Li et al., 2022). Among the elements related to plant growth and development, the TATA-box was the most common element in the promoter region of eukaryotic genes. The TATA-box is not only involved in initiating transcription machinery assembly but also indirectly involved in plant adaptation to environmental conditions such as light (Savinkova et al., 2023). Among the elements involved in phytohormone response, the largest number was ABRE, which is consistent with findings in wheat and maize. Overexpression of ABscisic acid response element-binding protein 1 (AREB1) in *A. thaliana* significantly upregulates the gene carrying ABRE motif and enhances drought tolerance in vegetative tissues (Barbosa et al., 2013).

The *EIN3/EIL* gene family is widely conserved across higher plants. Research has demonstrated that ethylene is capable of responding to various biotic/abiotic stresses, including drought and low temperature, and of enhancing plant tolerance by regulating gene expression. In *Medicago sativa*, *MsEIL1*, *MsEIL4*, and *MsEIL5* are involved in responses to drought, cold, and other stresses (Su et al., 2024). In broomcorn millet (*Panicum miliaceum* L.), *PmEIL3* and *PmEIL8* were significantly up-regulated in response to low-temperature stress (Yang et al., 2024). Overexpression of *MnEIL3* in *Arabidopsis* increased salt tolerance and drought tolerance (Liu et al., 2019). All these results suggest that the *EIN3/EIL* gene family plays an important role in abiotic stress. Taking advantage of this natural adaptation, we systematically dissected the expression characteristics of the *ScEILs* under low-temperature and drought stresses. The results indicate that low temperatures rapidly upregulate the expression of *ScEIL1* and *ScEIL2*, suggesting that these two genes may act as early responsive factors in rye perception of low temperature signals. Within 24 h of drought treatment, the expression of *ScEIL4* showed a unimodal increase, with a significantly higher amplitude than that of other members, suggesting that it plays a “dominant switch” role in the drought response module. Tissue-specific expression analysis further indicated that the expression of *ScEIL4* was relatively low under normal conditions but was sharply induced under drought stress, which conformed to the typical characteristics of a “stress-specific” regulator.

As a close wild relative of wheat, rye is a valuable genetic reservoir for disease resistance and stress tolerance. The successful introgression of the dominant stripe rust resistance gene *Yr9* from rye into wheat underscores its historical importance as a core donor for wheat improvement (Rabanus-Wallace et al., 2021). EIN3/EIL proteins are key transcription factors in the ethylene signaling pathway and have been implicated in pathogen defense across various plant species. In this study, we found that the expression of *ScEIL5* in rye was significantly induced at 48 hpi with stripe rust. Analysis of *cis-acting* elements revealed that the *ScEIL5* promoter is enriched with TC-rich repeats, which are associated with defense and stress responsiveness, suggesting a potential role for *ScEIL5* in biotic stress responses. Functional validation using VIGS confirmed this hypothesis. Silencing of *ScEIL5* led to a significant reduction in fungal growth, as evidenced by lower spore counts, fungal biomass, hyphal length, and infection area compared to the BSMV:γ control. Furthermore, DAB staining revealed a substantially larger area of H_2_O_2_ accumulation in *ScEIL5*-silenced leaves, indicating a strengthened oxidative burst. Collectively, these results demonstrate that *ScEIL5* negatively regulates rye’s resistance to stripe rust. This conclusion aligns with previous reports on certain *EIN3/EIL* family members, such as the negative regulator *TaEIL1* in wheat and the significant roles of group B EIN3/EIL proteins in disease resistance (He et al., 2020). Our study not only identifies *ScEIL5* as a biotic stress-responsive gene in rye but also provides new insights into the functional diversity of *EIN3/EIL* genes in stripe rust resistance. Therefore, the continued exploration of resistance genes in rye, coupled with advanced gene editing technologies, represents a promising strategy for developing wheat and related cereals with durable resistance to stripe rust.

## 5 Conclusion

In this study, we conducted genome-wide identification and characterization of the *EIN3/EIL* gene family in rye (*Secale cereale* L.) and identified seven *ScEIL* genes. Phylogenetic analysis revealed significant evolutionary divergence between monocotyledonous and dicotyledonous *EIN3/EIL* branches. Gene-structure analysis further indicated high conservation of the N-terminal region. Expression profiling showed that each *ScEIL* member responds to at least one environmental stress, highlighting their extensive involvement in biotic and abiotic stress-defence networks. VIGS-mediated silencing of *ScEIL5* reduced fungal biomass, enhanced stripe-rust resistance, and elevated H_2_O_2_ accumulation, demonstrating that *ScEIL5* negatively regulates stripe-rust resistance in rye.

## Data Availability

The datasets presented in this study can be found in online repositories. The names of the repository/repositories and accession number(s) can be found in the article/**Supplementary Material**.
